# A systematic review and meta-analysis: clinical outcomes of recurrent pregnancy failure resulting from preimplantation genetic testing for aneuploidy

**DOI:** 10.3389/fendo.2023.1178294

**Published:** 2023-10-02

**Authors:** Zhuo Liang, Qiuyue Wen, Jingjing Li, Dingyuan Zeng, Pinxiu Huang

**Affiliations:** ^1^ Center of Reproductive Medicine, Guangzhou Women and Children’s Medical Center-Liuzhou Hospital, Liuzhou, Guangxi, China; ^2^ Center of Reproductive Medicine, Liuzhou Maternal and Child Health Hospital, Liuzhou, Guangxi, China; ^3^ Center of Reproductive Medicine, Liuzhou Institute of Reproduction and Genetics, Liuzhou, Guangxi, China; ^4^ Affiliated Maternity Hospital and Affiliated Children’s Hospital of Guangxi, University of Science and Technology, Liuzhou, Guangxi, China; ^5^ Guangxi Clinical Research Center for Obstetrics and Gynecology, Liuzhou, Guangxi, China; ^6^ Graduate School, Guilin Medical College, Guilin, Guangxi, China

**Keywords:** preimplantation genetic testing for aneuploidy, recurrent pregnancy failure, meta-analysis, next genetic screening, blastocyst biopsy, aneuploidy

## Abstract

**Background:**

Preimplantation genetic testing for aneuploidy (PGT-A) is an emerging technology that aims to identify euploid embryos for transfer, reducing the risk of embryonic chromosomal abnormalities. However, the clinical benefits of PGT-A in recurrent pregnancy failure (RPF) patients, particularly in young RPF patients, remains uncertain.

**Objective and rationale:**

This meta-analysis aimed to determine whether RPF patients undergoing PGT-A had better clinical outcomes compared to those not undergoing PGT-A, thus assessing the value of PGT-A in clinical practice.

**Search methods:**

We systematically searched PubMed, the Cochrane Library, China National Knowledge Infrastructure (CNKI), Wanfang Data, and VIP Database for Chinese Technical Periodicals (VIP) from 2002 to 2022. Thirteen published studies involving 930 RPF patients screened using PGT-A and over 1,434 RPF patients screened without PGT-A were included in this meta-analysis. Clinical outcomes were evaluated based on embryo transfers after PGT-A (n=1,015) and without PGT-A (n=1,799).

**Clinical outcomes:**

The PGT-A group demonstrated superior clinical outcomes compared to the *in vitro* fertilization (IVF)/intracytoplasmic sperm injection (ICSI) group. The PGT-A group had a significantly higher implantation rate (IR) (RR=2.01, 95% CI: [1.73; 2.34]), clinical pregnancy rate (CPR) (RR=1.53, 95% CI: [1.36; 1.71]), ongoing pregnancy rate (OPR) (RR=1.76, 95% CI: [1.35; 2.29]), live birth rate (LBR) (RR=1.75, 95% CI: [1.51; 2.03]), and significantly lower clinical miscarriage rate (CMR) (RR=0.74, 95% CI: [0.54; 0.99]). Subgroup analysis based on patient age (under 35 years and 35 years or older) showed that both PGT-A subgroups had significantly better CPR (P<0.01) and LBR (P<0.05) values compared to the IVF/ICSI groups.

**Summary:**

This meta-analysis demonstrates that PGT-A in RPF patients, is associated with improved clinical outcomes, including higher IR, CPR, OPR, and LBR values, and lower CMR compared to the IVF/ICSI group. These findings support the positive clinical application of PGT-A in RPF patients.

**Systematic Review Registration:**

http://INPLASY.com, identifier INPLASY 202320118.

## 1 Introduction

Recurrent spontaneous abortion (RSA) and recurrent implantation failure (RIF) are common conditions associated with recurrent pregnancy failure (RPF). RSA is defined as two or more spontaneous abortions in a female with the same sexual partner (Gardner et al., 1999; Capalbo et al., 2014) (1, 2). RIF refers to the failure to achieve a clinical pregnancy after transfer of at least four good-quality embryos in a minimum of three fresh or frozen cycles in a woman under the age of 40 years (Coughlan C., 2014) (3). Notably, embryonic chromosomal abnormalities are the most frequent cause of early pregnancy failure, with aneuploidy being the most common chromosomal abnormality (Lee et al., 2015) (4). Preimplantation genetic testing for aneuploidy (PGT-A) is a clinical screening method that aims to identify euploid embryos, thereby reducing the risks of implantation failure and pregnancy loss due to embryonic chromosomal abnormalities. It is recommended for females of advanced age, as well as those with RSA or RIF (PGD/PGS techniques expert consensus writing group 2018) (5). However, PGT-A has associated risks and ethical challenges, such as high costs, difficulties in culturing embryos from advanced-age patients to the standard blastocyst biopsy stage, potential embryo damage or loss, and embryo wastage (Liu et al., 2016) (6). The clinical benefits of PGT-A in RPF patients remain controversial. In light of this, our study aimed to explore the value of PGT-A in RPF patients to provide insights for its clinical application.

## 2 Theory and method

This meta-analysis adhered to the PRISMA guidelines, and the study was registered with INPLASY (http://INPLASY.com) under registration number INPLASY 202320118.

### 2.1 Data search

We conducted a systematic search of the following databases from 2002 to 2022: PubMed, the Cochrane Library, CNKI, Wanfang Data, and VIP. Two researchers (Liang, Z., and Wen, Q. Y.) performed the search using advanced retrieval techniques. The key terms used in the search included recurrent miscarriage (RM), recurrent spontaneous abortion (RSA), recurrent implantation failure (RIF), *in vitro* fertilization (IVF), intracytoplasmic sperm injection (ICSI), preimplantation genetic screening (PGS), preimplantation genetic testing for aneuploidy (PGT-A), next genetic screening (NGS), array-based comparative genomic hybridization (aCGH), fluorescence *in situ* hybridization (FISH), blastocyst biopsy, and aneuploidy.

### 2.2 Study selection

The studies were initially selected and independently reselected by two researchers (Liang, Z., and Wen, Q.Y.) based on their titles and abstracts. The inclusion criteria for the initial selection were as follows: (1) The study belonged to the discipline of medicine; (2) It was a published Chinese or English study, excluding conference studies or case studies; (3) The study design involved a controlled study with an IVF/ICSI control group; (4) The study focused on human subjects; and (5) The article included raw study data. Subsequently, the reselection process included the following criteria: (1) The studied patients had each experienced two or more spontaneous abortions each; (2) Each patient under the age of 40 years had received at least 4 good-quality cleavage embryos in a minimum of three fresh or frozen cycles without achieving pregnancy; and (3) Neither the patients nor their husbands had chromosomal abnormalities. Exclusion criteria were also applied, which were: (1) Patients diagnosed with exceptional immunity, including antiphospholipid syndrome; (2) Patients diagnosed with anatomical abnormalities of the genital tract through gynecological ultrasound, salpingography, or laparoscopy; (3) Patients who used donated oocytes or donated sperm; (4) Patients diagnosed with endocrine abnormalities; (5) Patients with prethrombotic conditions; (6) Patients who had reproductive tract infections; (7) Patients whose husbands had semen abnormalities as determined by semen examination; (8) Patients with life risk factors for pregnancy loss; (9) Patients who had not achieved live births but still had available embryos. In cases where there was controversy regarding the final selection decision, consensus was reached through discussion with a third researcher (Huang, P.X.).

### 2.3 Data extraction

Two researchers (Liang, Z., and Wen, Q.Y.) independently extracted data from the reselected studies. After extracting the data, we carefully compared the two data sets; if discrepancies emerged, a third researcher (Huang, P.X.) determined how to proceed. The extracted data included information such as the first author, year of publication, study design, study period, sample details, patient characteristics, and outcomes. Primary clinical outcomes, including implantation rate (IR), clinical pregnancy rate (CPR), clinical miscarriage rate (CMR), ongoing pregnancy rate (OPR), and live birth rate (LBR), were also extracted.

### 2.4 Groups and subgroups

For this meta-analysis, we divided the RPF patients into two groups: a PGT-A group and an IVF/ICSI group. The division was made prior to extracting data on the patients’ primary clinical outcomes. As females age, their ovarian function declines, resulting in increased chromosomal variations in oocytes and aneuploidy rates in embryos, eventually stabilizing at approximately 85% (Alfarawati et al., 2011; Rabinowitz et al., 2012; Hassold et al., 2009; Harton et al., 2013; Dang et al., 2019; Rubio et al., 2003; Franasiak et al., 2014) (7–13) To explore the value of PGT-A in different age groups, we categorized the patients into two subgroups: those under 35 years of age and those 35 years of age or older. While there is evidence supporting the benefits of PGT-A for advanced-age RPF patients, it remains uncertain whether young RPF patients can benefit from PGT-A. Therefore, our focus was on determining whether PGT-A could improve the clinical outcomes of young RPF patients. Due to the limited number of studies included in our analysis, we only compared CLR, CMR, and LBR values among the subgroups.

### 2.5 Quality assessment

The quality assessments of the included studies were conducted independently by two researchers (Liang, Z., and Wen, Q.Y.). In case of any discrepancies, a third researcher (Huang, P.X.) determined the course of action. The Cochrane Handbook (Cumpston et al., 2019) was utilized to evaluate the quality of randomized controlled trials (RCTs) (14) was utilized to evaluate the quality of randomized controlled trials (RCTs). This evaluation encompassed random sequence generation, allocation concealment, blinding of outcome assessment, incomplete outcome data, selective reporting and other biases (15). Each item was assessed as low, high or unclear. To assess the quality of cohort studies, we employed the Risk of Bias in Non-randomized Studies of Interventions tool (Sterne et al., 2016) (15). This evaluation considered biases arising from confounding, selection of participants, classification of interventions, deviations from intended interventions, missing data, measurement of outcomes and selection of the reported results (16). Each study was categorized as having low, moderate, serious, critical or unclear risk of bias.

### 2.6 Statistical analysis

We utilized R version 4.2.1 for statistical analysis in this meta-analysis. The results were presented as relative ratios (RRs) with corresponding 95% confidence intervals (CIs). An RR value greater than 1 indicated a positive correlation, while a value less than 1 indicated a negative correlation. Heterogeneity was assessed using the Q test and I^2^ statistics, and H-statistics were calculated Q-statistics. Due to the limited number of primary outcomes included in our analysis, we considered Q-statistics’ p-values of less than 0.10 as statistically significant. We simultaneously evaluated the H-statistics and the I^2^ statistics. H-statistics equal to 1 indicated no heterogeneity, H-statistics less than 1.2 indicated negligible heterogeneity, and H-statistics greater than 1.5 indicated heterogeneity. For H-statistics ranging from 1.2 to 1.5, heterogeneity was indicated by 95% CIs excluding 1, while inclusion of 1 in the 95% CIs made heterogeneity uncertain. I^2^ statistics greater than 50% indicated high heterogeneity. Subsequently, we conducted a sensitivity analysis to identify heterogeneity by individually excluding studies in an attempt to reduce the I^2^ statistics to less than 50%. Since we could not accurately obtain the data that may have influenced heterogeneity, such as racial differences, we could not analyze the sources of the heterogeneity in this meta-analysis. Therefore, we employed a random effects model for all outcomes. Additionally, as we included more than 10 studies in certain comparisons, we conducted a publication bias analysis using Egger analysis, considering the likelihood of positive data being published more than negative data. For all other analysis results, P-values less than 0.05 were considered statistically significant differences. The following calculations using data were performed on primary clinical outcomes: IR = (pregnant fetuses ÷ total transplantation cycles) × 100%; CPR = (clinical pregnancy cycles ÷ total transplantation cycles) × 100%; CMR = clinical miscarriage cycles ÷ clinical pregnancy cycles × 100%; OPR = ongoing pregnancy cycles ÷ total transplantation cycles × 100%; LBR = live birth cycles ÷ total transplantation cycles × 100%.

## 3 Results

### 3.1 Results of searches

Two researchers independently conducted searches in PubMed, the Cochrane Library, CNKI, Wanfang Data, and VIP made starting in 2002 and ending in 2022. After the initial selection and reselection process, we included fourteen studies in our analysis (Cheng et al., 2022; Kong et al., 2021; Li et al., 2019; Murugappan et al., 2016; Dai 2020; Yang et al., 2019; Sui et al., 2020; Rubio et al., 2013; Fodina et al., 2021; Pantou et al., 2022; Sato et al., 2019; Ma et al., 2020; Zhang et al., 2018; Blockeel et al., 2008) (16–29).

### 3.2 Results of quality assessment

Among the four randomized controlled trials (RCTs) included (Sui et al., 2020; Rubio et al., 2013; Sato et al., 2019; Blockeel et al., 2008), we found a high risk of bias in the blinding of participants and personnel, as well as an unclear risk in blinding of outcome assessment bias item. Only one study (Sato et al., 2019) had an unclear risk of bias in the incomplete outcome data, while the remaining studies had a high risk. The other bias items were assessed as low risk. We excluded one study (Blockeel et al., 2008) as its inclusion would have impacted the results. Regarding the cohort studies, four studies were assessed as low risk of bias, six studies as moderate risk, and one as high risk. The results of the quality assessment are presented by us (**Supplementary Figure S1**, **Table S2**).

### 3.3 Characteristics of the included studies

**Figure 1:**
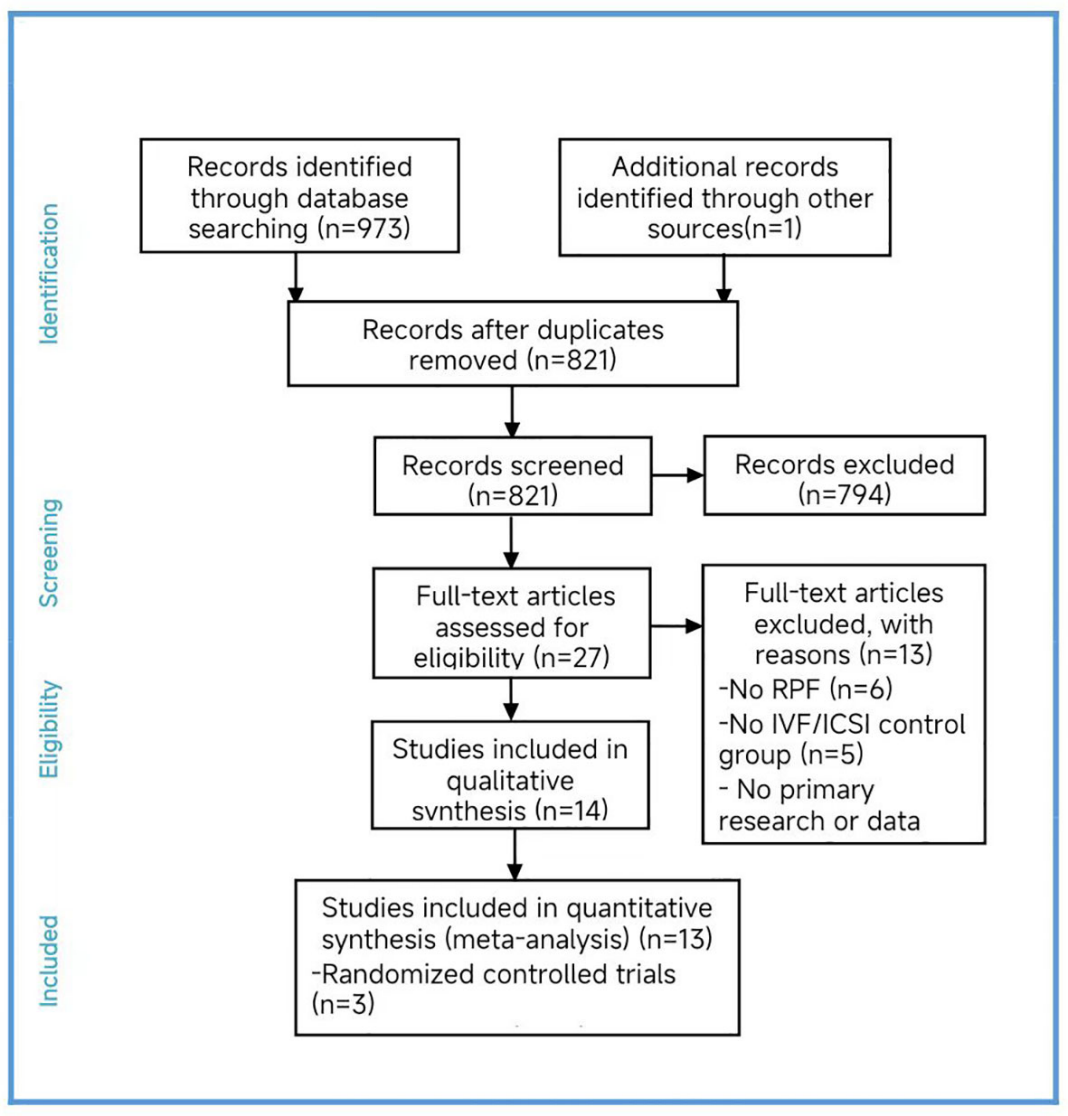
PRISMA flow chart.

In total, we included thirteen published studies in this meta-analysis, involving 930 recurrent pregnancy failure (RPF) patients screened using PGT-A and over 1,434 RPF patients screened without PGT-A. The PGT-A group had 1,015 embryo transfers, while the IVF/ICSI group had 1,799 embryo transfers (**Table 1**). We analyzed six studies in the implantation rate (IR) group, thirteen studies in the clinical pregnancy rate (CPR) group, twelve studies in the clinical miscarriage rate (CMR) group, four studies in the ongoing pregnancy rate (OPR) group, and ten studies in the live birth rate (LBR) group (**Table 2**). Additionally, we categorized six studies by age, including three studies in the young group and five studies in the advanced-age group (**Table 3**). Thirteen studies were excluded after full-text screening, and one study was excluded after quality assessment (**Supplementary Table S1**). The entire screening process was shown by us (**Figure 1**).

**Table 1:** Characteristics of included studies.

Author (year)	Study design	Sample details			Population		Outcomes included in this analysis	Over judgement of study quality
		The biopsy stage	Fresh or frozen embryo transfer	Testing methods	Experimental group	Control group		
Cheng, H., et al. (2022) (16)	Cohort study	Blastocyst	PGT-A: frozen IVF/ICSI: Fresh/frozen	NGS	39 singletons	Fresh: 46 (11 singletons and 35 multiples) Frozen: 113 singletons	IR, CPR, CMR, and OPR	Moderate risk
Kong, N. N., et al. (2021) (17)	Cohort study	Blastocyst	Frozen	NGS	66 singletons	23 singletons	CPR, CMR, and LBR	Low risk
Li, Y. Q., et al. (2019) (18)	Cohort study	Blastocyst	PGT-A: frozen IVF/ICSI: fresh	NGS	34 singletons	51 singletons	CPR, CMR	Moderate risk
Murugappan, G., et al. (2016) (19)	Cohort study	Blastocyst	PGT-A: frozen IVF/EM: fresh	aCGH	64 singletons	102 singletons	CPR, CMR, and LBR	Low risk
Dai, X. (2020) (20)	Cohort study	Blastocyst	PGT-A: frozen IVF/ICSI: fresh	NCS	132 singletons	564 singletons	CPR, CMR, and LBR	Moderate risk
Yang, J. W., et al. (2019) (21)	Cohort study	Blastocyst	PGT-A: frozen IVF/ICSI: fresh	NGS	55 singletons	40 singletons	IR, CPR, and CMR	Low risk
Sui, Y.L., et al. (2020) (22)	RCT	Blastocyst	PGT-A: frozen IVF/ICSI: fresh	NGS	103 singletons/multiples	104 singletons/multiples	IR, CPR, CMR, OPR, and LBR	N/A
Rubio, C., et al. (2013) (23)	RCT	Cleavage	PGS: frozen IVF/ICSI: fresh	FISH	48 singletons/multiples	43 singletons/multiples	IR, CPR, CMR, OPR, and LBR	N/A
Pantou, A., et al. (2022) (25)	Cohort study	Blastocyst	PGT-A: frozen IVF/ICSI: fresh	aCGH	RIF: 30 RM: 25 singletons/multiples	RIF: 42 RM: 40 singletons/multiples	IR, CPR, CMR, and LBR	Moderate risk
Sato, T., et al. (2019) (26)	RCT	Blastocyst	PGT-A: frozen IVF/ICSI: fresh	aCGH	RM: 41 RIF: 42 singletons	RM: 38 RIF: 50 singletons	CPR, CMR, and LBR	N/A
Ma, H. P., et al. (2020) (27)	Cohort study	Blastocyst	PGT-A: frozen IVF/ICSI: fresh	NGS	92 singletons	N/A	CPR and LBR	Moderate risk
Zhang, D. D., et al. (2018) (28)	Cohort study	Blastocyst	PGT-A: frozen IVF/ICSI: frozen	aCGH	72 singletons	68 singletons	IR, CPR, CMR, and LBR	Moderate risk
Fodina, V., et al. (2021) (24)	Cohort study	Blastocyst	PGT-A: frozen IVF/ICSI: frozen	NGS	87 singletons	72 singletons	CPR and CMR	Low risk

**Table 2:** Pooled meta-analysis results.

Group	No. of studies	No. of events/total	Effect model	Effect size (RR [95 CI%])	P-value	I^2^ (%)
IR	6	PGT-A: 213/452 IVF/ICSI: 247/1010	Random	2.01 [1.73; 2.34]	< 0.0001	0.0%
CPR	12	PGT-A: 464/785 IVF/ICSI: 645/1597	Random	1.53 [1.36; 1.71]	< 0.0001	31.4%
CMR	11	PGT-A: 70/497 IVF/ICSI: 148/653	Random	0.74 [0.55; 1.00]	0.0473	19.9%
OPR	4	PGT-A: 143/292 IVF/ICSI: 130/445	Random	1.76 [1.35; 2.89]	< 0.0001	42.7%
LBR	9	PGT-A: 323/648 IVF/ICSI: 434/1438	Random	1.75 [1.51; 2.03]	< 0.0001	41.8%

**Table 3:** Pooled results of analyses for subgroups.

Subgroups		No. of studies	No. of events/total	Effect model	Effect size (RR [95 CI%])	P-value	I^2^ (%)
Young group	CPR	3	PGT-A: 115/185 IVF/ICSI: 211/395	Random	1.23 [1.07; 1.42]	0.0041	0.0%
	CMR	3	PGT-A: 18/115 IVF/ICSI: 35/211	Random	0.98 [0.52; 1.86]	0.9554	5.1%
	LBR	2	PGT-A: 85/170 IVF/ICSI: 149/344	Random	1.22 [1.01; 1.49]	0.0401	0.0%
Advanced-age group	CPR	4	PGT-A: 103/162 IVF/ICSI: 204/538	Random	2.05 [1.73; 2.43]	< 0.0001	0.0%
	CMR	4	PGT-A: 26/135 IVF/ICSI: 62/221	Random	0.80 [0.51; 1.24]	0.3222	15.4%
	LBR	5	PGT-A: 138/262 IVF/ICSI: 230/840	Random	1.86 [1.59; 2.18]	< 0.0001	0.0%

### 3.4 Clinical outcomes of the PGT-A and IVF/ICSI groups

#### 3.4.1 Implantation rate

**Figure 2:**
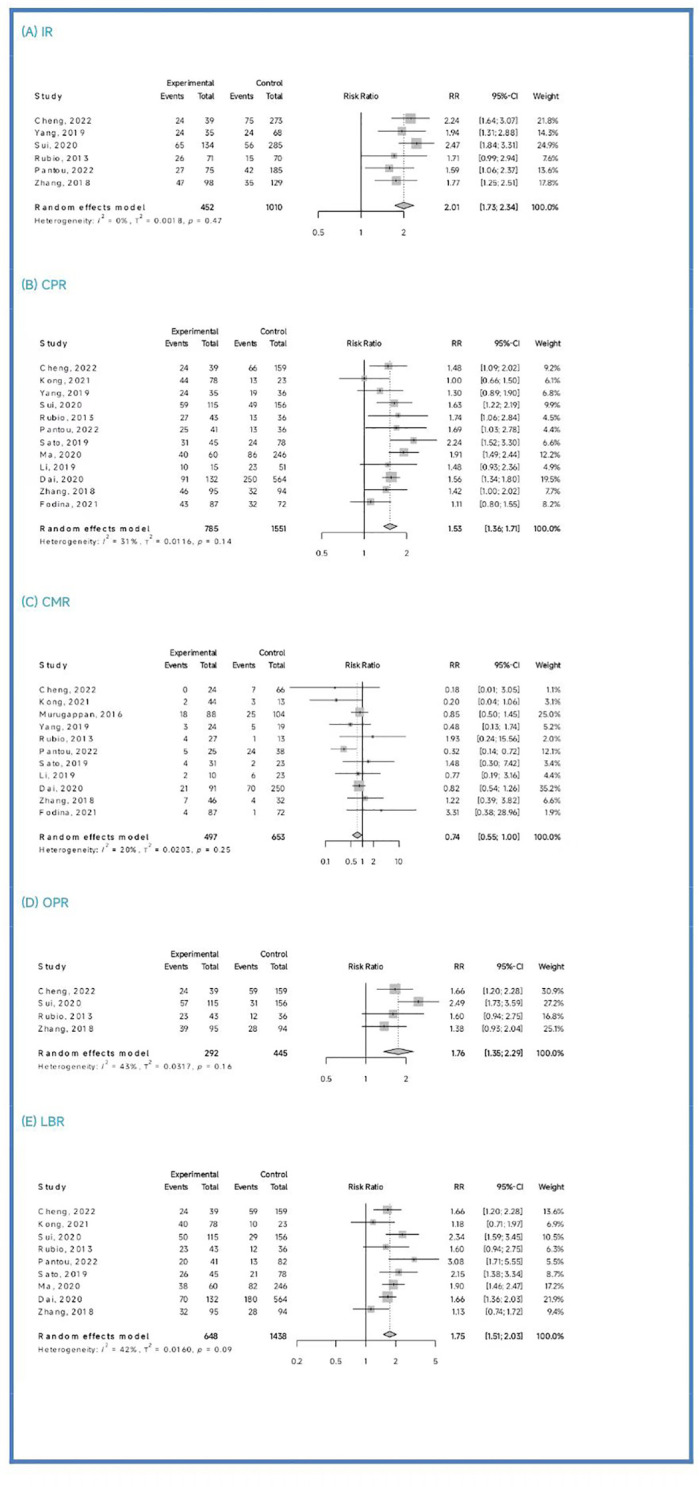
Pooled meta-analysis results.

Among the six studies (Cheng et al., 2022; Yang et al., 2019; Sui et al., 2020; Rubio et al., 2013; Pantou et al., 2022; Zhang et al., 2018) included in this analysis, the PGT-A group showed a significantly higher IR than the IVF/ICSI group (RR = 2.01, 95% CI: [1.73; 2.34], z = 9.05, P < 0.00001, I^2 = ^0.0%, H = 1.00, **Table 2**, **Figure 2A**).

#### 3.4.2 Clinical pregnancy rate

Out of the thirteen initially included studies, we excluded one study (Murugappan, et al., 2016) after performing a sensitivity analysis and temporarily excluding each study to identify the source of the high bias. Eventually, we included twelve studies for analysis and found no significant publication bias (p = 0.63 > 0.05). The CPR was significantly higher in the PGT-A group compared to IVF/ICSI group (RR = 1.53, 95% CI: [1.36; 1.71], z = 7.32, P < 0.0001, I^2 = ^31.4%, H = 1.21 [1.00; 1.70], **Table 2**, **Figure 2B**).

#### 3.4.3 Clinical miscarriage rate

Out of the twelve studies initially included (Cheng et al., 2022; Kong et al., 2021; Murugappan et al., 2016; Dai 2020; Yang et al., 2019; Rubio et al., 2013; Fodina et al., 2021; Pantou et al., 2022; Sato et al., 2019; Ma et al., 2020; Zhang et al., 2018; Blockeel et al., 2012), we excluded one study (Sui, et al., 2020) after conducting a sensitivity analysis and temporarily excluding each study to determine the source of the high bias. Thus, a total of eleven studies were included before conducting an analysis of publication bias, which indicated no significant publication bias (p = 0.86 > 0.05). The CMR was significantly decreased in the PGT-A group than in the IVF/ICSI group (RR = 0.74, 95% CI: [0.54; 1.00], z = −1.98, P = 0.047 < 0.05, I^2 = ^19.9%, H = 1.12 [1.00; 1.57], **Table 2**, **Figure 2C**).

#### 3.4.4 Ongoing pregnancy rate

Among the four studies (Cheng et al., 2022; Sui et al., 2020; Rubio et al., 2013; Zhang et al., 2018) included in this analysis, the OPR was significantly higher in the PGT-A group compared to the IVF/ICSI group (RR = 1.76, 95% CI: [1.36; 2.29], z = 4.21, P < 0.0001, I^2 = ^42.7%, H = 1.32 [1.00; 2.28], **Table 2**, **Figure 2D**).

#### 3.4.5 Live birth rate

After performing a sensitivity analysis and temporarily excluding each study to determine the source of the high bias, we finally included nine studies (Cheng et al., 2019; Kong et al., 2021; Murugappan et al., 2016; Dai 2020; Sui et al., 2020; Rubio et al., 2013; Pantou et al., 2022; Sato et al., 2019; Ma et al., 2020; Zhang et al., 2018), and excluded one studies (Murugappan, et al., 2016). The LBR was significantly higher in the PGT-A group compared to the IVF/ICSI group (RR = 1.75, 95% CI: [1.51; 2.03], z = 7.35, P < 0.0001, I^2 = ^41.8%, H = 1.31 [1.00; 1.93], **Table 2**, **Figure 2E**).

### 3.5 Clinical outcomes of PGT-A group and IVF/ICSI group in the young subgroup

#### 3.5.1 Clinical pregnancy rate

**Figure 3:**
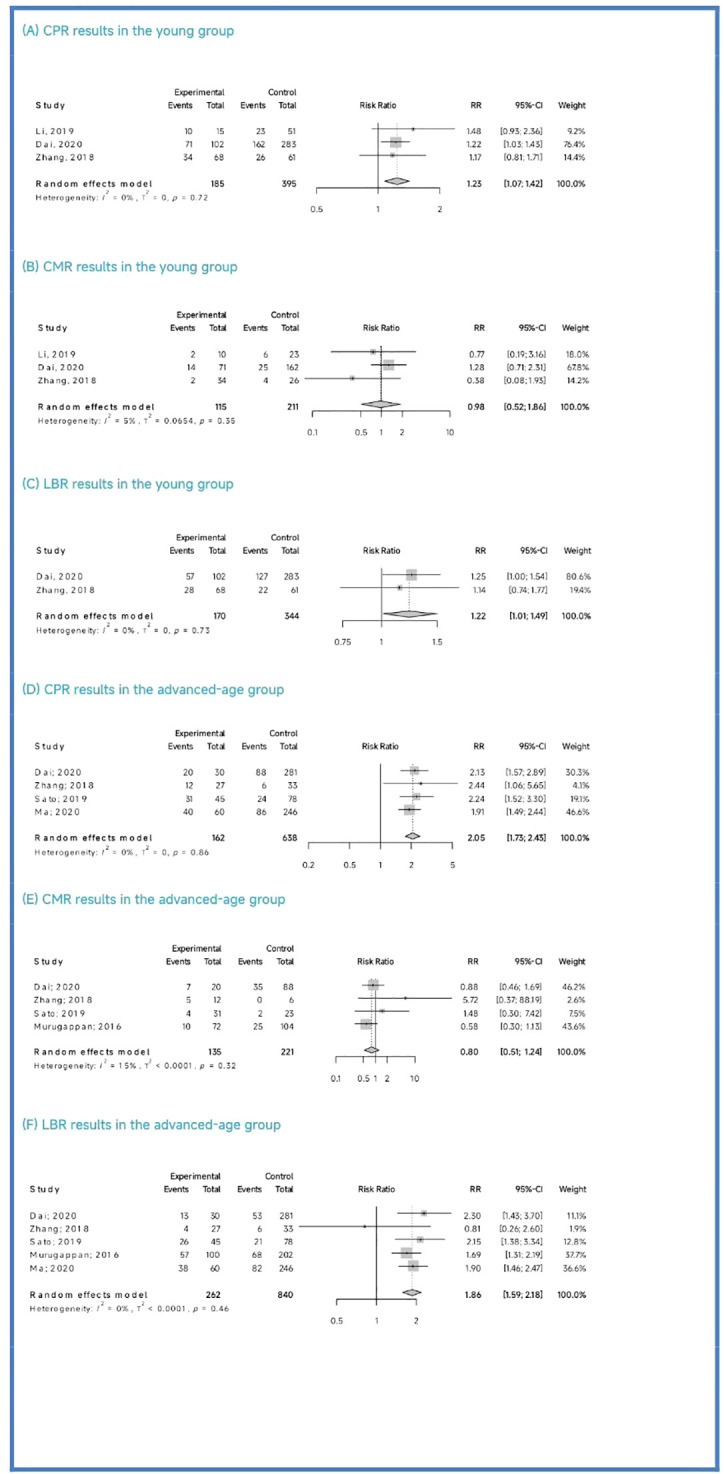
Pooled analysis results for subgroups.

Regarding the CPR, among the three included studies (Li et al., 2019; Dai 2020; Zhang et al., 2018), the CPR was significantly higher in the PGT-A group than in the IVF/ICSI group (RR = 1.23, 95% CI: [1.07; 1.42], z = 2.87, P = 0.0041 < 0.05, I^2 = ^0%, H = 1.00, **Table 3**, **Figure 3A**).

#### 3.5.2 Clinical miscarriage rate

Regarding the CMR, based on the three included studies (Li et al., 2019; Dai 2020; Zhang et al., 2018), there was no significant difference between the PGT-A and IVF/ICSI groups in terms of CMR, but the CMR showed a downward trend in the PGT-A group (RR = 0.98, 95% CI: [0.51; 1.85], z = −0.06, P = 0.9554 > 0.05, I^2 = ^5.1%, H = 1.03, **Table 3**, **Figure 3B**).

#### 3.5.3 Live birth rate

Regarding the LBR, only two studies (Dai 2020; Zhang et al., 2018) were included in this analysis, and the LBR was significantly higher in the PGT-A group than in the IVF/ICSI group (RR = 1.22, 95% CI: [1.01; 1.49], z = 2.05, P = 0.0401 < 0.05, I^2 = ^0%, H = 1.00, **Table 3**, **Figure 3C**).

### 3.6 Clinical outcomes of the PGT-A and IVF/ICSI groups in the advanced-age subgroup

#### 3.6.1 Clinical pregnancy rate

Among the five studies (Murugappan et al., 2016; Dai 2020; Sato et al., 2019; Ma et al., 2020; Zhang et al., 2018) initially included, one study (Murugappan, et al., 2016) was excluded after performing a sensitivity analysis and temporarily excluding each study to determine the source of the high variability in the studies. Ultimately, four studies were included, and the CPR was significantly higher in the PGT-A group compared to the IVF/ICSI group (RR = 2.05, 95% CI: [1.73; 2.43], z = 8.36, P < 0.0001, I^2 = ^0%, H = 1.00, **Table 3**, **Figure 3D**).

#### 3.6.2 Clinical miscarriage rate

Regarding the CMR, we included four studies (Murugappan et al., 2016; Dai 2020; Sato et al., 2019; Zhang et al., 2018) in this analysis, and there was no significant difference between the PGT-A and IVF/ICSI groups in terms of CMR (RR = 0.80, 95% CI: [0.51; 1.24], z = −0.99, P = 0.3222 > 0.05, I^2 = ^15.4%, H = 1.09, **Table 3**, **Figure 3E**).

#### 3.6.3 Live birth rate

Regarding the LBR, we included five studies (Murugappan et al., 2016; Dai 2020; Sato et al., 2019; Ma et al., 2020; Zhang et al., 2018) in this analysis, and the LBR was significantly higher in the PGT-A group compared to the IVF/ICSI group (RR = 1.86, 95% CI: [1.59; 2.18], z = 7.67, P < 0.0001, I^2 = ^0.0%, H = 1.00, **Table 3**, **Figure 3F**).

## 4 Discussion

PGT-A is a novel genetic screening method that is gaining clinical significance due to continuous advancements in theory and technology. Researchers are increasingly exploring the potential benefits of PGT-A in improving clinical outcomes.

Our analysis on whether RPF patients can benefit from PGT-A has yielded conflicting results. Some studies support the notion that PGT-A can enhance clinical outcomes in RPL patients, while others (Hodes et al., 2012; Perfetto et al., 2015) suggest that PGT-A does not contribute to improvement and may even worsen clinical outcomes (30, 31). In this meta-analysis, we have examined the available data in the context of the provided background and offered insights into the clinical application of PGT-A.

After evaluating all thirteen published studies, we observed that in the examined RPF patients, the PGT-A group exhibited higher rates of IR, CPR, OPR, and LBR, along with a lower rate of CMR compared to the IVF/ICSI group. Furthermore, upon stratifying the RPF patients into subgroups based on age, we found that the PGT-A group demonstrated significant improvements in CPR and LBR.

Our meta-analysis revealed significant advantages in the clinical outcomes of studied RPF patients who underwent PGT-A. We identified early indications of the benefits associated with the clinical application of PGT-A. For instance, Yang’s study (Yang et al., 2012) proposed that the PGT-A group would exhibit significantly better CPR and OPR values compared to the IVF/ICSI group (32). Subsequently, additional evidence from various researchers emerged, supporting the advantages of PGT-A. Neal’s study (Neal et al., 2018) demonstrated improved IR and reduced CMR with PGT-A (33). While Liang’s study (Liang et al., 2020) revealed enhancements in CPR and LBR (34). After 2021, an increasing number of studies on PGT-A were published. Sadecki’s study (Sadecki et al., 2021) suggested an improvement in LBR with PGT-A (35). Bhatt’s study (Bhatt et al., 2021) indicated potential improvements in CPR, CMR, and LBR with PGT-A (36). While Kato’s study (Kato et al., 2021) also suggested enhanced LBR and reduced CMR (37). Considering the results of this meta-analysis, it is evident that RPF patients can enhance their clinical outcomes by utilizing PGT-A.

Regarding the benefits of employing PGT-A for screening advanced-age patients, Mastenbroek’s earlier studies (Mastenbroek et al., 2007) (Mastenbroek et al., 2011) suggested a significant reduction in OPR and LBR through preimplantation genetic screening (PGS) (38, 39). A controversy regarding the ability of PGT-A’s clinical application to provide benefits was sparked. However, the findings of these two studies have become relatively irrelevant due to various factors, such as technological advancements and embryo biopsy occurring during the cleavage stage (Scott et al., 2011) (40). Sacchi’s study (Sacchi et al., 2019) demonstrated a significant improvement in LBR and reduction in CMR for advanced-age patients using PGT-A (41). Similarly, Murphy (Murphy et al., 2019) and Lee (Lee et al., 2019) reported significant improvements in LBR for advanced-age patients through PGT-A (42, 43). Furthermore, Munné’s study (Munné et al., 2019) revealed a significant improvement in OPR with PGT-A (44). In 2022, Hao (Hao et al., 2022) provided further evidence of significant enhancements in CPR and LBR, accompanied by a notable reduction in CMR for advanced-age patients (45). These results strongly indicate favorable clinical outcomes resulting from the use of PGT-A. Therefore, ample evidence suggests that advanced-age RPF patients can enhance their clinical outcomes through the application of PGT-A. In this analysis, we found that PGT-A significantly improved CPR and LBR while decreasing CMR in advanced-age patients. As patients age, the quality of their oocytes declines, leading to increased chromosomal variations and higher rates of embryo aneuploidy. Embryonic chromosomal abnormalities are a common cause of recurrent pregnancy failure, making advanced-age patient potential beneficiaries of PGT-A.

The potential benefits of preimplantation genetic testing for aneuploidy (PGT-A) in young patients remain uncertain due to limited research in this area. Murphy’s study (Murphy et al., 2019) suggested that young patients may not derive any advantages from PGT-A and, in fact, it could even reduce their LBR (42). This viewpoint was supported by Yan’s study (Yan et al., 2021) (46). However, these two studies had limitations in terms of their patient selection. Murphy’s study focused solely on young patients, while Yan’s study included women with good fertility who may not have had indications for PGT-A. Therefore, the findings of these studies have limited applicability. In our analysis, we specifically examined patients with a history of RPF, which is a highly complex condition in young patients that cannot be fully addressed by PGT-A alone. Nonetheless, we found that in young RPF patients, PGT-A significantly improved CPR, LBR, and decreased CMR, indicating the potential for improvement in this group.

We also investigated the influence of different PGT-A methods across different age groups. Our findings indicated that both the aCGH group and the NGS group demonstrated higher CPR and LBR values compared to the IVF/ICSI group in advanced-age patients. However, in young patients, aCGH did not provide benefits, whereas NGS significantly improved CPR and LBR (**Supplementary Tables S2, S3**; **Supplementary Figure S3, S4**).

Of course, further research is warranted to elucidate the value of PGT-A, considering the limited number of studies available in the subgroups we analyzed. Additionally, the influence of various factors, such as technological advancements in PGT-A, the choice between frozen or fresh embryos (Wong et al., 2017; Rodrigue et al., 2016; Roque et al., 2013), single-embryo or multiple-embryo transfer (Gleicher et al., 2017), study design, embryonic damage, mosaic blastocyst discard (Liu et al., 2021), and the accuracy of trophectoderm biopsy, should be taken into account when interpreting the results of this meta-analysis (47–51).

Looking ahead, we anticipate that more studies will emerge on the clinical application of PGT-A in young RPF patients, further elucidating the value of this screening method.

## 5 Strengths and limitations

In this meta-analysis, we conducted a systematic search of studies published between 2002 and 2022, strictly selecting them based on our predetermined criteria, and subsequently performed an analysis. This comprehensive analysis of PGT-A in the context of RPF can serve as a valuable reference for the improvement and application of PGT-A.

However, it is important to note that this analysis has some limitations, including the following: (1) We included a relatively small number of thirteen studies, and only six studies could be included in subgroup analyses due to limited publications; and (2) the available raw data were not comprehensive enough, which hindered our ability to perform source analysis regarding potential factors that may have contributed to heterogeneity (e.g., racial differences, the distinction between single-embryo transfer and multiple-embryo transfer).

## 6 Conclusion

In conclusion, our meta-analysis revealed that PGT-A has the potential to improve IR, CPR, OPR, and LBR, while reducing CMR in RPF patients. These findings indicate favorable clinical outcomes associated with PGT-A screening. Furthermore, our results suggest that advanced-age patients can benefit from PGT-A, potentially may reducing the time to achieve live births (Zhao et al., 2019; Rubio et al., 2017) (52, 53). Taking into consideration the complex etiology of young patients, it should be noted that PGT-A may not address all of their underlying issues. Nevertheless, we recommend considering PGT-A for young RPF patients, considering the significant psychological pressure and substantial economic burden they often face. It is worth mentioning that although our analysis indicates benefits for young patients, further large-scale controlled trials are needed to further support our conclusions drawn from this meta-analysis.

## Data availability statement

Existing datasets are available in a publicly accessible repository: Publicly available datasets were analyzed in this study. This data can be found here: [10.6084/m9.figshare.24148338].

## Author contributions

Conception and design: DZ, JL, and PH. Collection and assembly of data: ZL, QW, and PH. Provision of study materials: ZL and QW. Data analysis and interpretation: ZL and PH. Manuscript writing: ZL. Administrative support: PH. All authors contributed to the article and approved the submitted version.
