# Supplementary figures and images for: A systematic review and meta-analysis: clinical outcomes of recurrent pregnancy failure resulting from preimplantation genetic testing for aneuploidy
Source: Front Endocrinol (Lausanne). 2023 Oct 2;14:1178294. doi: 10.3389/fendo.2023.1178294 (PMC10577404; doi:10.3389/fendo.2023.1178294)

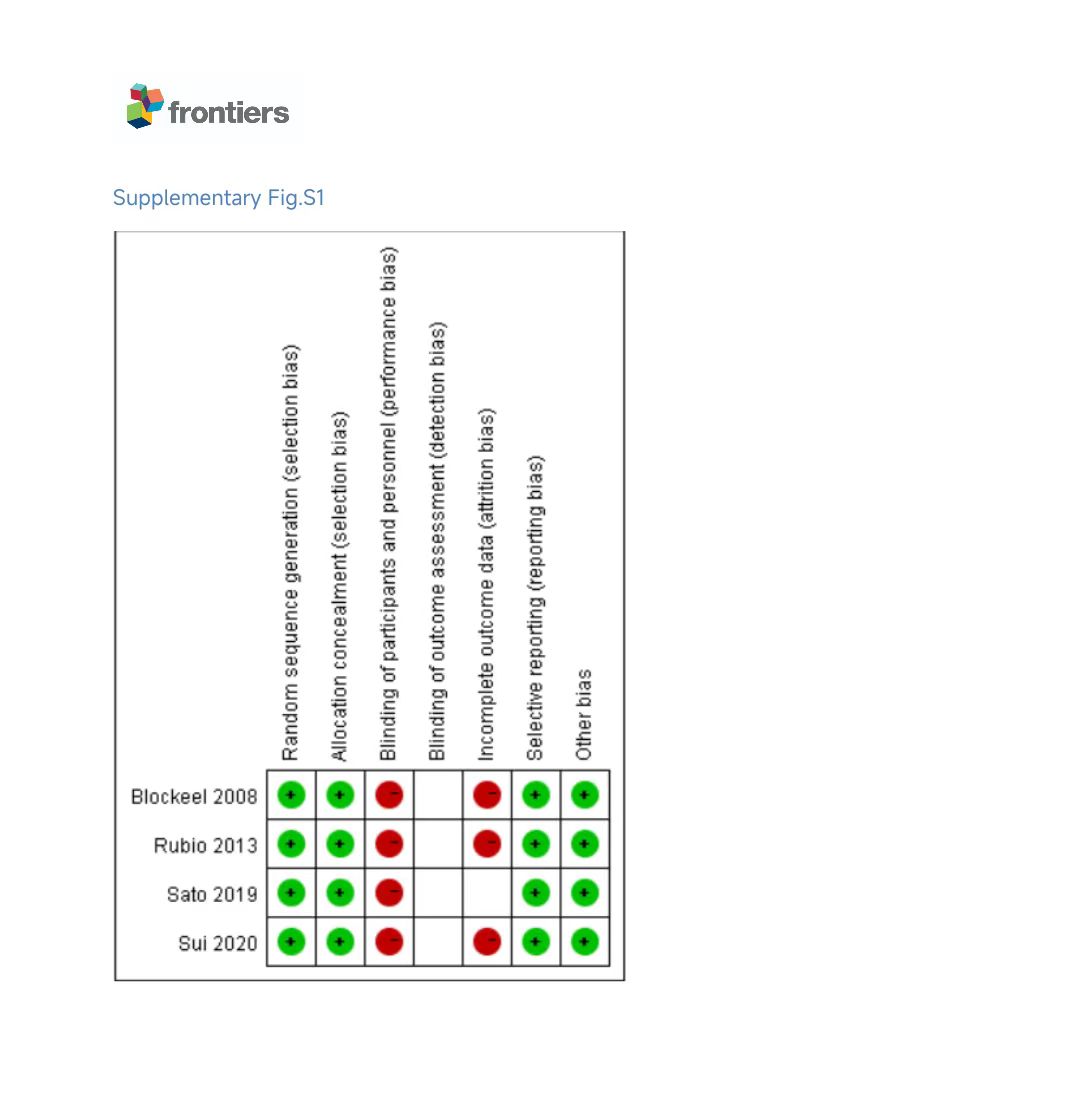

Supplement: Supplementary file 1 [file Image_1.jpg]

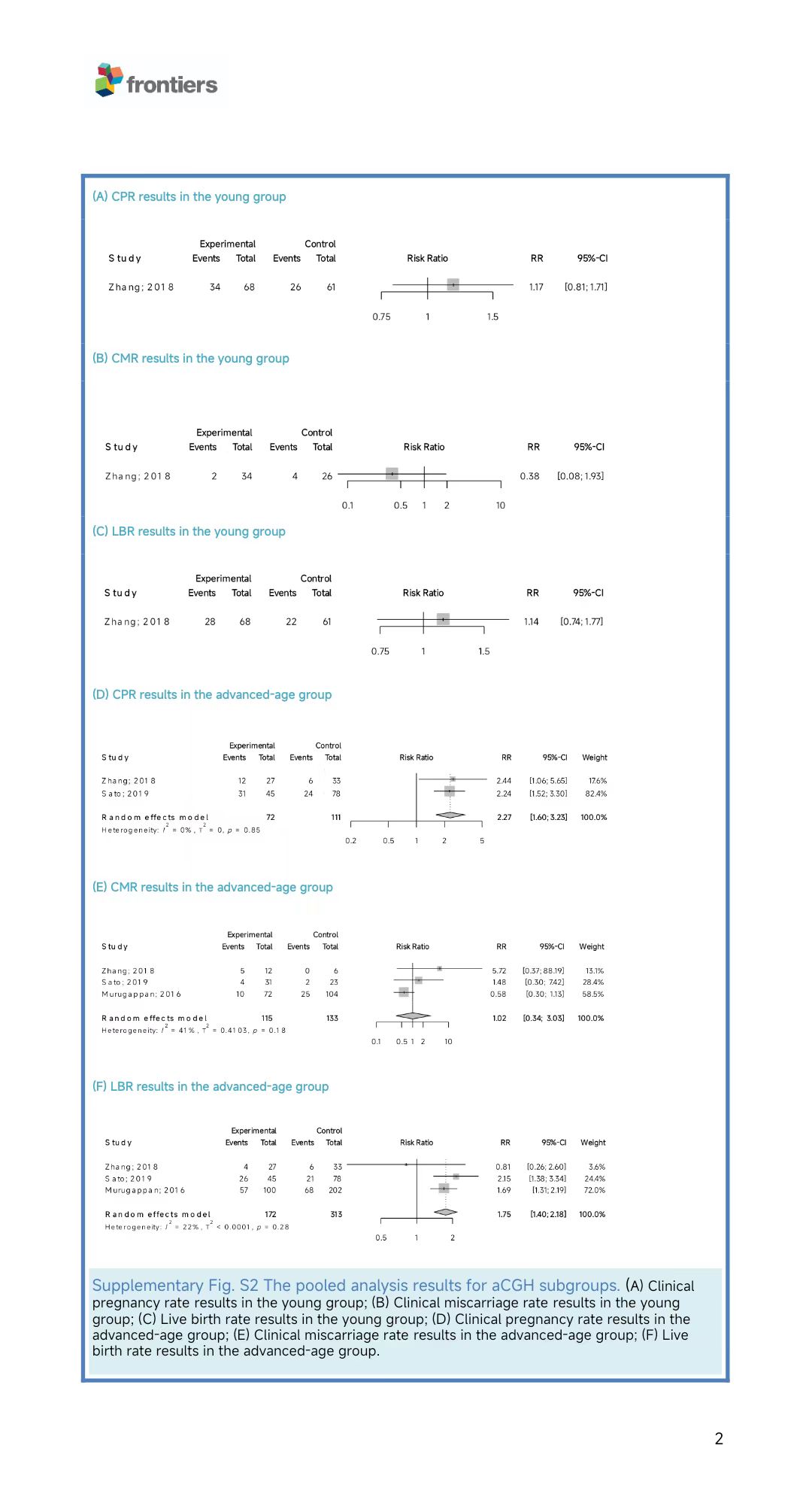

Supplement: Supplementary file 2 [file Image_2.jpg]

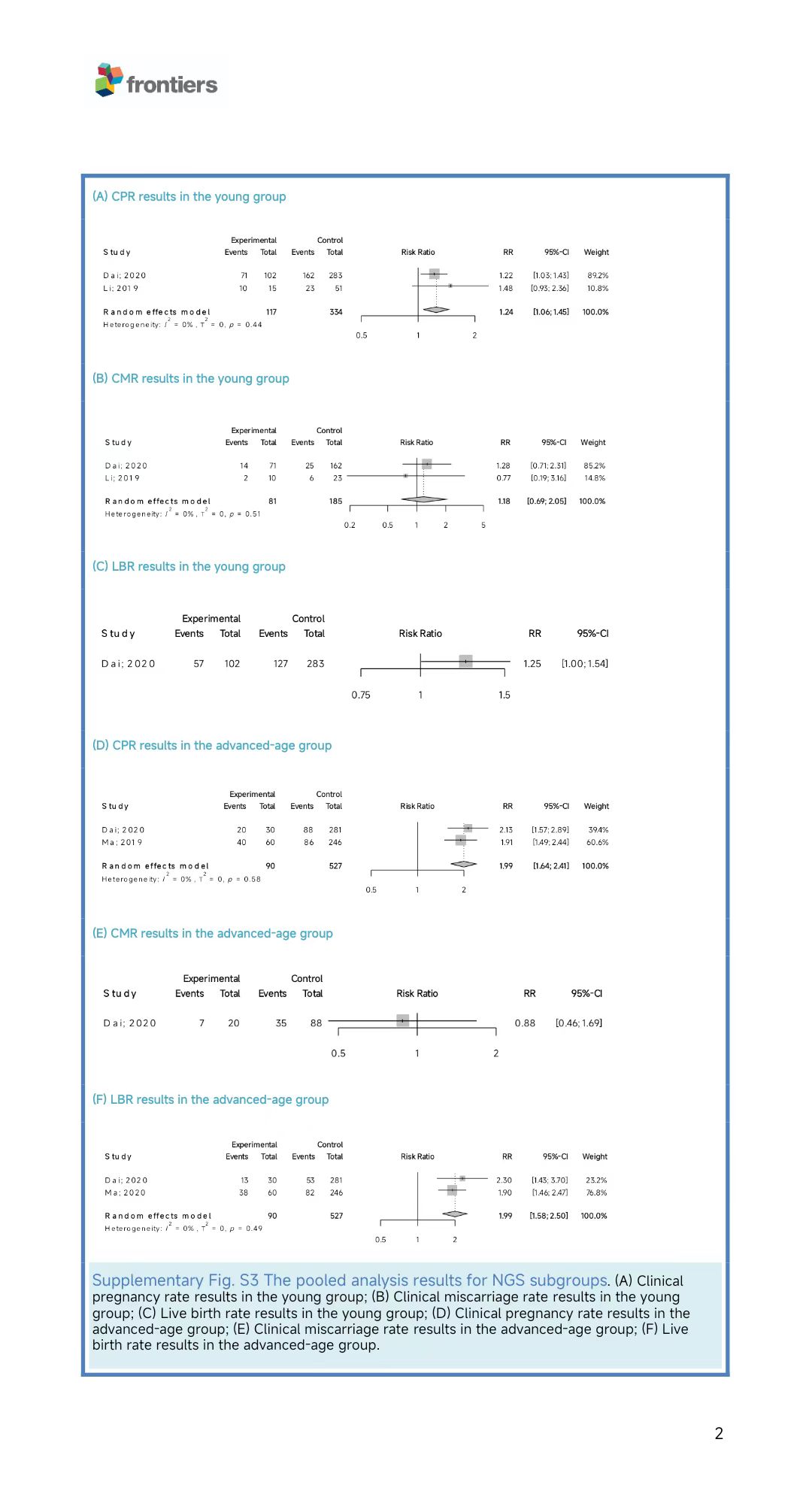

Supplement: Supplementary file 3 [file Image_3.jpg]
