# Supplementary material for: A systematic review and meta-analysis: clinical outcomes of recurrent pregnancy failure resulting from preimplantation genetic testing for aneuploidy
Source: Front Endocrinol (Lausanne). 2023 Oct 2;14:1178294. doi: 10.3389/fendo.2023.1178294 (PMC10577404; doi:10.3389/fendo.2023.1178294)
Supplement: Supplementary file 4 [file Table_1.docx]

Supplementary Table S1 Excluded studies

| Author (year) | Title | Reasons for exclusion |
| --- | --- | --- |
| Liu et al. (2019) | Pregnancy outcome analysis of preimplantation genetic testing for women of advanced maternal age. | No RPF |
| Bo et al. (2017) | Effect of biopsy timing and detection method of PGD/PGS on pregnancy outcomes. | No IVF/ICSI control group |
| Liang et al. (2020) | Comparison of pregnancy outcomes of frozen embryo transfer in ICSI/PGT-ART. | No RPF |
| Song et al. (2022) | Application of PGT-A and PGT-SR in assisted reproduction. | No IVF/ICSI control group |
| Tong et al. (2021) | Next-Generation Sequencing (NGS)-Based Preimplantation Genetic Testing for Aneuploidy (PGT-A) of Trophectoderm Biopsy for Recurrent Implantation Failure (RIF) Patients: a Retrospective Study. | No IVF/ICSI control group |
| Yan et al. (2021) | Live Birth with or without Preimplantation Genetic Testing for Aneuploidy. | No RPF |
| Mastenbroek et al. (2007) | In vitro fertilization with preimplantation genetic screening. | No RPF |
| Rubio et al. (2017) | In vitro fertilization with preimplantation genetic diagnosis for aneuploidies in advanced maternal age: a randomized, controlled study. | No RPF |
| Han et al. (2018) | Application of preimplantation genetic screening in recurrent spontaneous abortion and elderly patients. | No IVF/ICSI control group |
| Huang et al. (2020) | Application value of preimplantation genetic screening (PGS) in elderly and recurrent spontaneous abortion (RSA) patients. | No IVF/ICSI control group |
| Leng et al. (2017) | Comparison of pregnancy outcome after D5 and D6 blastocysts under freeze-thawing in two kinds of ART. | No primary research or data |
| Sadecki et al. (2021) | Comparison of live birth rates after IVF-embryo transfer with and without preimplantation genetic testing for aneuploidies. | No primary research or data |
| Hao et al. (2022) | Maternal and perinatal outcomes after preimplantation genetic testing for aneuploidies using blastocyst biopsy for women of advanced age. | No RPF |
| Blockeel et al. (2008) | Prospectively randomized controlled trial of PGS in IVF/ICSI patients with poor implantation. | High risk of bias items |
