# Supplementary material for: A systematic review and meta-analysis: clinical outcomes of recurrent pregnancy failure resulting from preimplantation genetic testing for aneuploidy
Source: Front Endocrinol (Lausanne). 2023 Oct 2;14:1178294. doi: 10.3389/fendo.2023.1178294 (PMC10577404; doi:10.3389/fendo.2023.1178294)
Supplement: Supplementary file 5 [file Table_2.docx]

Supplementary Table S2 Risk of bias summary.

| Aim: | | To assess the effect of assignment to intervention, | | | | | | | |
| --- | --- | --- | --- | --- | --- | --- | --- | --- | --- |
| Outcomes: | | The clinical outcomes like implantation rate, clinical pregnancy rate, clinical miscarriage rate, ongoing pregnancy rate and live birth rate. | | | | | | | |
| Important confounding factors: | | Demographics, methods of genetic testing, single-embryo transfer or multiple-embryo transfer, the selection of frozen or fresh embryos. | | | | | | | |
| Author  (year) | Bias due to confounding  (Support for judgement) | | Bias in selection of participants into study  (Support for judgement) | Bias in classification of interventions  (Support for judgement) | Bias due to deviation from intended intervention  (Support for judgement) | Bias due to missing data  (Support for judgement) | Bias in measurement of outcomes  (Support for judgement) | Bias in selection of the reported result  (Support for judgement) | Overall Judgement |
| Cheng, H., et al.  (2022) | Low risk  (The study adjusted for important confounding domains) | | Low risk  (The participants in this study include the population we want to evaluate) | Low risk  (The intervention was defined as embryo biopsy, which was very clear) | Low risk  (During the study period, the intervention received by the participants in the study group and the control group was fixed) | Moderate risk  (The proportion of participants of intervention group (97%) and control group (79%) was moderate) | Low risk  (The observed outcomes were objectively and were not easy to produce the measurement bias) | Low risk  (This is a complete cohort study and the results were all reported in the article) | Moderate risk |
| Kong, N. N., et al.  (2021) | Low risk  (The study adjusted for important confounding domains) | | Low risk  (The participants in this study include the population we want to evaluate) | Low risk  (The intervention was defined as embryo biopsy, which was very clear) | Low risk  (During the study period, the intervention received by the participants in the study group and the control group was fixed) | Low risk  (The outcomes data were available for all participants in this study) | Low risk  (The observed outcomes were objectively and were not easy to produce the measurement bias) | Low risk  (This is a complete cohort study and the results were all reported in the article) | Low risk |
| Li, Y. Q., et al.  (2019) | Moderate risk  (Although the author matches some baseline data, there are still some confounding factors that the author cannot control) | | Low risk  (The participants in this study include the population we want to evaluate) | Low risk  (The intervention was defined as embryo biopsy, which was very clear) | Low risk  (During the study period, the intervention received by the participants in the study group and the control group was fixed) | Low risk  (The proportion of missing data was very small, and the proportion of participants for missing data comparable across interventions | Low risk  (The observed outcomes were objectively and were not easy to produce the measurement bias) | Low risk  (This is a complete cohort study and the results were all reported in the article) | Moderate risk |
| Murugappan, G., et al.  (2016) | Low risk  (The study adjusted for important confounding domains) | | Low risk  (The participants in this study include the population we want to evaluate) | Low risk  (The intervention was defined as embryo biopsy, which was very clear) | Low risk  (During the study period, the intervention received by the participants in the study group and the control group was fixed) | Low risk  (The outcomes data were available for all participants in this study) | Low risk  (The observed outcomes were objectively and were not easy to produce the measurement bias) | Low risk  (This is a complete cohort study and the results were all reported in the article) | Low risk |
| Dai, X.  (2020) | Low risk  (The authors used multivariable regression model to adjusted the important confounding domains for main outcomes) | | Low risk  (The participants in this study include the population we want to evaluate) | Low risk  (The intervention was defined as embryo biopsy, which was very clear) | Low risk  (During the study period, the intervention received by the participants in the study group and the control group was fixed) | Moderate risk  (The proportion of participants of intervention group (83%) and control group (86%) was moderate) | Low risk  (The observed outcomes were objectively and were not easy to produce the measurement bias) | Low risk  (This is a complete cohort study and the results were all reported in the article) | Moderate risk |
| Yang, J. W., et al.  (2019) | Low risk  (The study adjusted for important confounding domains) | | Low risk  (The participants in this study include the population we want to evaluate) | Low risk  (The intervention was defined as embryo biopsy, which was very clear) | Low risk  (During the study period, the intervention received by the participants in the study group and the control group was fixed) | Low risk  (The outcomes data were available for all participants in this study) | Low risk  (The observed outcomes were objectively and were not easy to produce the measurement bias) | Low risk  (This is a complete cohort study and the results were all reported in the article) | Low risk |
| Pantou, A., et al.  (2022) | Moderate risk  (Although the author matches some baseline data, there are still some confounding factors that the author cannot control) | | Low risk  (The participants in this study include the population we want to evaluate) | Low risk  (The intervention was defined as embryo biopsy, which was very clear) | Low risk  (During the study period, the intervention received by the participants in the study group and the control group was fixed) | Low risk  (The outcomes data were available for all participants in this study) | Low risk  (The observed outcomes were objectively and were not easy to produce the measurement bias) | Low risk  (This is a complete cohort study and the results were all reported in the article) | Moderate risk |
| Ma, H. P., et al.  (2020) | Moderate risk  (The authors did not adjust or match the important confounding factors) | | Low risk  (The participants in this study include the population we want to evaluate) | Low risk  (The intervention was defined as embryo biopsy, which was very clear) | Low risk  (During the study period, the intervention received by the participants in the study group and the control group was fixed) | Low risk  (The outcomes data were available for all participants in this study) | Low risk  (The observed outcomes were objectively and were not easy to produce the measurement bias) | Low risk  (This is a complete cohort study and the results were all reported in the article) | Moderate risk |
| Zhang, D. D., et al.  (2018) | Moderate risk  (The authors did not adjust or match the important confounding factors) | | Low risk  (The participants in this study include the population we want to evaluate) | Low risk  (The intervention was defined as embryo biopsy, which was very clear) | Low risk  (During the study period, the intervention received by the participants in the study group and the control group was fixed) | Low risk  (The outcomes data were available for all participants in this study) | Low risk  (The observed outcomes were objectively and were not easy to produce the measurement bias) | Low risk  (This is a complete cohort study and the results were all reported in the article) | Moderate risk |
| Fodina, V., et al.  (2021) | Low risk  (The study adjusted for important confounding domains) | | Low risk  (The participants in this study include the population we want to evaluate) | Low risk  (The intervention was defined as embryo biopsy, which was very clear) | Low risk  (During the study period, the intervention received by the participants in the study group and the control group was fixed) | Low risk  (The outcomes data were available for all participants in this study) | Low risk  (The observed outcomes were objectively and were not easy to produce the measurement bias) | Low risk  (This is a complete cohort study and the results were all reported in the article) | Low risk |
