# Supplementary material for: A systematic review and meta-analysis: clinical outcomes of recurrent pregnancy failure resulting from preimplantation genetic testing for aneuploidy
Source: Front Endocrinol (Lausanne). 2023 Oct 2;14:1178294. doi: 10.3389/fendo.2023.1178294 (PMC10577404; doi:10.3389/fendo.2023.1178294)
Supplement: Supplementary file 6 [file Table_3.docx]

| Supplementary Table S3 The pooled results of analyses for aCGH subgroups | | | | | | | |
| --- | --- | --- | --- | --- | --- | --- | --- |
| The aCGH subgroups | | No. of studies | No. of events/total | Effect model | Effect size (RR [95 CI%]) | P-value | I^2^ (%) |
| Young group | CPR | 1 | PGT-A: 34/68  IVF/ICSI: 26/61 | Random | 1.17 [0.81; 1.71] | 0.4052 | N/A |
|  | CMR | 1 | PGT-A: 2/34  IVF/ICSI: 4/26 | Random | 0.38 [0.08; 1.93] | 0.2444 | N/A |
|  | LBR | 1 | PGT-A: 28/68  IVF/ICSI: 22/61 | Random | 1.14 [0.74; 1.77] | 0.5537 | N/A |
| Advanced-age group | CPR | 2 | PGT-A: 43/72  IVF/ICSI: 30/111 | Random | 2.27 [1.60; 3.23] | < 0.0001 | 0.0% |
|  | CMR | 3 | PGT-A: 19/115  IVF/ICSI: 27/133 | Random | 1.02 [0.34; 3.03] | 0.9725 | 41.0% |
|  | LBR | 3 | PGT-A: 87/172  IVF/ICSI: 95/313 | Random | 1.75 [1.40; 2.18] | < 0.0001 | 21.6% |
