# Supplementary material for: A systematic review and meta-analysis: clinical outcomes of recurrent pregnancy failure resulting from preimplantation genetic testing for aneuploidy
Source: Front Endocrinol (Lausanne). 2023 Oct 2;14:1178294. doi: 10.3389/fendo.2023.1178294 (PMC10577404; doi:10.3389/fendo.2023.1178294)
Supplement: Supplementary file 7 [file Table_4.docx]

| Supplementary Table S4 The pooled results of analyses for NGS subgroups | | | | | | | |
| --- | --- | --- | --- | --- | --- | --- | --- |
| The NGS subgroups | | No. of studies | No. of events/total | Effect model | Effect size (RR [95 CI%]) | P-value | I^2^ (%) |
| Young group | CPR | 2 | PGT-A: 81/117  IVF/ICSI: 185/334 | Random | 1.24 [1.06; 1.45] | 0.0058 | 0.0% |
|  | CMR | 2 | PGT-A: 16/81  IVF/ICSI: 31/185 | Random | 1.18 [0.69; 2.05] | 0.5435 | 0.0% |
|  | LBR | 1 | PGT-A: 57/102  IVF/ICSI: 127/283 | Random | 1.25 [1.00; 1.54] | 0.0460 | N/A |
| Advanced-age group | CPR | 2 | PGT-A: 60/90  IVF/ICSI: 174/527 | Random | 1.99 [1.64; 2.41] | < 0.0001 | 0.0% |
|  | CMR | 1 | PGT-A: 7/20  IVF/ICSI: 35/88 | Random | 0.88 [0.46; 1.69] | 0.7000 | N/A |
|  | LBR | 2 | PGT-A: 52/90  IVF/ICSI: 135/527 | Random | 1.99 [1.58; 2.50] | < 0.0001 | 0.0% |
